# Poly(ADP-Ribose) Polymerase 1: Cellular Pluripotency, Reprogramming, and Tumorogenesis

**DOI:** 10.3390/ijms160715531

**Published:** 2015-07-09

**Authors:** Bo-Hua Jiang, Wei-Lien Tseng, Hsin-Yang Li, Mong-Lien Wang, Yuh-Lih Chang, Yen-Jen Sung, Shih-Hwa Chiou

**Affiliations:** 1 Institute of Oral Biology, National Yang-Ming University, Taipei 112, Taiwan; E-Mail: lovelace831124@yahoo.com.tw; 2 School of Medicine, National Yang-Ming University, Taipei 112, Taiwan; E-Mails: jan740709@gmail.com (W.-L.T.); doc3643h@yahoo.com.tw (H.-Y.L.); monglien@gmail.com (M.-L.W.); ylchang@vghtpe.gov.tw (Y.-L.C.); yjsung@ym.edu.tw (Y.-J.S.); 3 Department of Medical Research, Taipei Veterans General Hospital, Taipei 112, Taiwan; 4 Institute of Anatomy and Cell Biology, School of Medicine, National Yang-Ming University, Taipei 112, Taiwan; 5 Department of Obstetrics and Gynecology, Taipei Veterans General Hospital, Taipei 112, Taiwan; 6 VGH-YM Genomic Research Center, National Yang-Ming University, Taipei 112, Taiwan; 7 Department of Pharmacy, Taipei Veterans General Hospital, Taipei 112, Taiwan

**Keywords:** Parp1, PARylation, pluripotency, reprogramming, tumorogenesis

## Abstract

Poly(ADP-ribos)ylation (PARylation) is the catalytic function of the Poly(ADP-ribose) polymerases (Parps) family for post-translational modification in cellular process. Being a major member of Parps, Parp1 is a crucial nuclear factor with biological significance in modulating DNA repair, DNA replication, transcription, DNA methylation and chromatin remodeling through PARylation of downstream proteins. In addition, high expression level and activity of Parp1 are correlated with pluripotent status, reprogramming, and cancer. Furthermore, epigenetic modulation of Parp1 is explored for regulating wide variety of gene expression. Genetic and pharmaceutical disruption of Parp1 further confirmed the importance of Parp1 in cell growth, DNA repair, and reprogramming efficiency. Taken together, the proximity toward the understanding of the modulation of Parp1 including interaction and modification in different fields will provide new insight for future studies. In this review, the biological significance of Parp1 in transcription and the epigenetic modulation of Parp1 in pluripotent status, reprogramming process and cancer will be summarized.

## 1. Introduction

Poly(ADP-ribose) polymerase 1 (Parp1), a multifunctional nuclear protein, is the major member of Parps family to promote poly(ADP-ribose) (PAR) chain formation through DNA-dependent modulation. This process of PAR chain formation is called poly(ADP-ribos)ylation (PARylation), a process that transfers a ADP-ribose group from nicotinamide adenine dinucleotide (NAD^+^) to the target protein, and changes the physical and enzymatic property of the target protein [1,2]. Because of the activity of PARylation, Parp1 is also called ADP-ribosyl transferase 1 (ARTD1). Parp1 was identified as a crucial modulator in DNA damage response in early studies [3,4]. However, other research shows that Parp1 also regulates protein function in cytoplasm [5,6,7]. Parp1 was first identified in malignant lymphoma to enhance the expression of poly(ADP-ribose) synthetase gene as well as the expressions of proto-oncogenes such as c-myc, c-fos, and c-myb. As a result, prior investigations aimed to address the role of Parp1 in tumorigenesis [8]. As a highly expressed protein in tumors, Parp1 modulated the DNA repair mechanism against genotoxic stress of mutagens [9]. However, recent studies showed that Parp1 does not only regulate DNA repair, but also plays various roles to modulate cell mitosis, DNA replication, gene expression, metabolism, and epigenetic events [3,4,10,11,12,13,14].

In order to reveal the biological significance of Parp1, a Parp1-null mouse model was established in 1995. Parp1-null mice are healthy and fertile, but these mice have high incidence of skin disease compared with wild-type mice [15]. In a subsequent study conducted in 2003, De Murcia *et al.* [16] reported that double-knockout of Parp1 and Parp2, another important member of the Parp family, causes embryonic lethality. Their results show Parp1 and Parp2 are essential to maintain genomic stability. Furthermore, Parp1 has also been revealed as haploinsufficiency to modulate centrosome duplication by PARylation while centrosome maintains chromosome stability [12].

Parp1 is highly expressed in pluripotent cells [17,18]. Deletion of Parp1 in mouse embryonic fibroblasts (MEF) results in lower efficiency of cell reprogramming [17]. Based on the above-mentioned results, Parp1 therefore affects reprogramming by modulating transcription, epigenetic events and chromatin stability. In this paper, we will discuss the mechanism involved in Parp1-driven pluripotency, cell reprogramming, and cancer.

## 2. Parp1, a Major Protein that Regulates PARylation in Cellular Process

### 2.1. Protein Structure of Parp1

Because Parp1 is the most abundant protein in the cell nucleus after histones, the function and mechanism of Parp1 have been investigated in many studies [2]. Parp1 has three functionally defined domains [19]: (1) N-terminal DNA binding domain; (2) C-terminal catalytic domain and (3) central automodification domain. The N-terminus of Parp1 contains DNA binding domain to recognize DNA break by zine-fingers structure, and this domain also regulates the catalytic activity of Parp1. In programmed cell death, caspase cleaves the N-terminal of Parp1 to block catalytic activity of Parp1 [20]. Of note, the loss of N-terminus decreases NAD^+^ consumption upon excess activation of PARylation leading to low energy in the cell. This negative feedback loop reveals the role of Parp1 in metabolism modulation. In DNA replication process, N-terminal domain also interacts with noncoding RNA and regulates the silent rRNA genes [21]. The C-terminus of Parp1 is a catalytic domain which binds to NAD^+^ and promotes PARylation of target proteins. Because of the functional importance of C-terminus, small molecules binding with the C-terminus of Parp1 are designed to block Parp1 activity as anticancer agents [22]. Parp1 is also modulated by post-translation modification (PTM) like PARylation and phosphorylation on the central automodification domain. The central automodification domain contains the BRCT (BRCA1 C-terminus) fold which mediates protein-to-protein interactions upon DNA repair, so Parp1 is suggested to be in charge of DNA repair [19]. PARylation activity of Parp1 is blocked by bridging integrator 1 (Bin1) which binds to automodification domain of Parp1 [23]. Interestingly, this regulation is modulated by c-Myc while c-Myc suppresses gene expression of Bin1 [24]. More regulations of Parp1 are being investigated in recent studies which provide more insights to the biological significance of Parp1 in epigenetic regulation and post-translational modification in cellular damages, as well as during cellular reprogramming and the maintenance of pluripotency.

### 2.2. PARylation Modulates Protein-to-Protein Interaction

PARylation is one ADP-ribose-transfer reaction for protein modification through the Parp family in response to DNA damage, chromatin-structure modulation, and cell division. PAR is also called third nucleic acid because it consists of adenosine and phosphate like DNA and RNA. Although similar to DNA and RNA, PAR does not have the ability to store chemical information during cellular process. The polymer of PAR contains several points of branching with 20–25 residues per branch, and this process provides a variable length of PAR from 2 to 200 units [25]. PARylation is commonly modified at the COOH residue of glutamates and aspartates in target proteins, and the automodification domain of Parp1 contains several residues as putative acceptors for the PAR chain [14,26]. The high molecular weight and negative charge of PAR affects protein properties such as structure and binding affinity [26]. PARylation also affects other post-translational modifications of target proteins like ubiquitination. A previous report demonstrated that PAR could prevent the ubiqitin-mediated proteasome degradation [27]. By specific affinity, Parp1 recruits associated proteins by PARylation for DNA repair and epigenetic modulation. A group of proteins such as MacroH2A contains PAR affinity domain (macro domain) to interact with PAR chain [13]. FACT (Facilitates Chromatin Transcription) complex is important in the cellular process to modulate DNA replication, and Parp1-mediated DNA repair mechanism through FACT complex is imperative. hSpt16, a subunit of the chromatin remodeling enzyme FACT complex, is PARylated during DNA repair response by Parp1 to modulate chromatin status. PARylation of hStp16 causes dissociation of FACT from chromatin [28]. Interestingly, both hSpt16 and Ssrp1 could be detected in pluripotent stem cells as PAR-associated proteins. The modulation of Ssrp1 through PARylation has also been confirmed in the reprogramming process [18]. Although PARylation contains biological significance, the amount of PARylation should be removed from the acceptors to maintain metabolic balance of NAD^+^. In order to regulate the cellular amount of PAR, Poly(ADP-ribose) glycohydrolase (Parg) degrades the PAR chains synthesized by Parp1 with low expression but with a high specific activity [29]. Fast turnover of PAR by Parg limits the half-life to less than 1 min under conditions of DNA breakage [1,30].

### 2.3. Multifunction of Parp1 in Transcriptional Regulation

In an early study of transcription regulation, Parp1 was identified with TFIIC to prevent RNA polymerase II from initiating transcription [31]. Growing evidence shows Parp1 plays a role in the modulation of gene expressions through chromatin modulation, enhancer binding, coregulation, and insulation [32]. These modulations are associated with the interaction between Parp1 and a wide variety of chromatin-associated proteins and transcription factors. In cell cycle modulation, Parp1 acts as the positive cofactor of E2F-1 during S-phase re-entry to regulate gene transcription, and the central automodification domain of Parp1 interacts with E2F-1 without direct PARylation at E2F-1 [11]. In fibrosis of tubular epithelial cells, the function of Parp1 for transcriptional regulation is a non-specific but fundamental enhancer of both basal and induced CCN2 gene transcription [33]. In epigenetic modulation, chromatin immunoprecipitation of Parp1 reveals the enrichment of Parp1 at the transcription start site with H3K4me3 and H3K9ac [34]. Parp1 also has the reciprocal binding ability with histone H1 at promoter sites while H1 works as a linking histone that causes compact chromatin structure [35]. In the nuclear reprogramming-mediated gene activation, Parp1 occupies gene loci of *Nanog* and *Esrrb*, affecting the recruitment of Oct4 and enrichment of H3K4me2 to promote gene activation [17].

T cell factor (TCF) 4 is an important modulator to β-catenin in Wnt pathway, which regulates the self-renewal in pluripotent stem cells and cell survival in cancer cells. By proteomic analysis, Parp1 is identified as a TCF4-associated protein and is essential for TCF4 downstream gene activation for proliferation in cancer cells. DNA damage mediated auto-PARylation of Parp1 suppresses the interaction between Parp1 and TCF4/β-catenin complex [36]. Furthermore, Ku70/Ku80 and Parp1 competitively affect the interaction of Parp1/TCF4/β-catenin in DNA damage response [37].

In CCCTC-binding factor (CTCF)-modulated DNA methylation, Parp1, and PARylation link the regulation between CTCF and Dnmt1 while CTCF activates PARylation through direct binding with Parp1 [38,39]. This epigenetic event is regulated by auto-PARylated Parp1 by CTCF mediated cross-talk between PARylation and DNA methylation. In another study, Parp1 is reported to localize at the Dnmt1 promoter to protect the unmethylated state by PARylation, and this modulation reveals Parp1 plays a role in epigenetic events by DNA methylation [40]. ATP-dependent remodeling enzyme modulates the functional state of chromatin, and nucleosome-remodeling ATPase ISWI genetically interacts with Parp [41]. Researchers therefore suggest Parp-mediated PARylation would regulate post-translational modification of ATP-dependent remodelers.

Nuclear respiratory factor 1 (NRF-1), a gene regulator of mitochondria biogenesis, also interacts with Parp1 and other Parp1 associated proteins like DNA-PK and Ku70 [42]. NRF-1 recruits Parp1 to the promoter site for transcriptional regulation. Parp1 simultaneously modifies the DNA binding domain of NRF-1 through PARylation. In short, Parp1 is involved in many mechanisms in the cellular process through direct or indirect transcription modulation.

## 3. Parp1 Plays an Essential Role in Pluripotent Status and Cell Reprogramming

### 3.1. The Potential Role of Parp1 in Pluripotency

A number of *in vivo* and *in vitro* studies on Parp 1 has reported knockout of Parp1 did not cause embryonic lethality in mouse embryogenesis [15]. Microarray analysis of Parp1-deficient embryonic stem cells (ESC) reveals the altered expression of genes involved in metabolism, signal transduction, and cell cycle [43]. These studies elucidate the important role of Parp1 in the maintenance of various genes on a genome-wild scale which is required to maintain pluripotency. However, a Parp family protein Parp2 might compensate the loss of Parp1 for pluripotency and embryogenesis, this compensation elucidates depletion of Parp1 in ESC would not have serious affects. Moreover, both Parp1 and Parp2 play a role in modulating endodermal differentiation through interacting with transcriptional intermediary factor beta (TIF1β) and the heterochromatin proteins (HP1) [44]. These results support the notion that Parp1 and Parp2 possess both non-redundant and overlapping functions in maintaining pluripotency.

### 3.2. Parp1 Maintains Pluripotent Status of Embryonic Stem Cells

Self-renewal is a crucial property of pluripotent stem cells in maintaining the undifferentiated state. By maintaining self-renewal, the stable transcription network should be activated without noise. Oct4, Sox2, and Nanog form a transcription network in pluripotent status to modulate gene expression [45]. Interestingly, Parp1 has been revealed as a protein that interacts with Oct4 and Sox2. The specific modulation between Parp1 and Oct4 has not been elucidated in pluripotent status, but Sox2 is PARylated by Parp1 for downstream gene activation in development [46,47,48,49]. PARylation of Sox2 has been explored in cell reprogramming to activate Fgf4 expression, and Fgf4 is essential for the first steps of reprogramming [49]. The importance of Parp-1-mediated PARylation of Sox2 supports that Parp1 is crucial for promoting cell reprogramming [17,18]. A loss-of-function study of Parg further shows that PARylation plays a crucial role for stem cell maintenance [50]. Telomere maintaining is another critical step in non-limited proliferation in pluripotent status. Inhibition or depletion of Parp1 leads to rapid shortening of telomere length without affecting the telomerase activity. This finding indicated that Parp1 plays a role in maintaining the telomere through PARylation [51]. It is interesting to note that although deletion of Parp1 is not sufficient to cause embryonic death, in combination with the deletion of other genes like Parp2, ku80, and DNA polymerase β shows relatively high rate of embryonic death [16,52,53]. Moreover, decreased Parp1 could attenuate neuronal apoptosis in adult mice [53]. Together, these results indicate that Parp1 is involved in several cellular processes in pluripotent status.

### 3.3. Parp1 and Cell Reprogramming

Cell reprogramming, also called nuclear reprogramming, is a process where somatic cells alter the gene expression pattern to pluripotent status. There are three approaches to achieve nuclear reprogramming: nuclear transfer, cell fusion, and transcription-factor transduction [54]. Transcription-factor transduction-mediated cell reprogramming was established in 2006 by Yamanaka Laboratory, and resulteing ES-like cells are called induced pluripotent stem (iPS) cells [55]. Transcription-factor transduction is a new technology in nuclear reprogramming; its’ efficiency and safety is crucial for further use in cell therapy. Previous studies following the establishment of transduction-mediated cell reprogramming focused on the quality and quantity of iPS cells. Latterly, several studies have emerged to investigate the mechanism induced by the defined transcription factors and the epigenetic events of reprogramming to establish safe and useful iPS cells [56]. The safety of iPS cells has improved in recent years with clinical trials started in some countries [57]. Cell reprograming also provides a technical platform in the investigation of gene regulation through epigenetic events like DNA methylation and histone modification. Parp1 is identified in cell reprogramming as c-Myc downstream gene, and has the potential to regulate PAR associated proteins like Sox2 [18,49]. Parp1 regulates cell reprogramming through transcription regulation or epigenetic modulation in the early stage of reprogramming. Sox2 is also modulated by Parp1 in the differentiation process to activate FGF4 expression [47,49]. As an Oct4 interacting protein, Parp1 participates in Tet2-mediated DNA methylation in cell reprogramming at several gene loci like *Nanog* and *Esrrb*. Moreover, the enrichment of H3K4me2 is affected by Parp1 in the reprogramming process, and this modulation reveals the correlation between Parp1 and the gene activation marker [17]. Histone modifications reveal different epigenetic events in gene regulation, and histone markers are remodeled in cell reprogramming. For instance, Wdr5 interacts with Oct4 and co-occupies gene loci of stemness genes in cell reprogramming for H3K4me enrichment while Wdr5 functions as an effector of H3K4 methylation [58]. By maintaining H3K4 methylation at gene loci, Parp1 could modulate KDM5B activation by PARylation and thereby affects chromatin structure by suppressing linking of Histone H1 [34]. In 2009, Chromodomain helicase DNA binding protein 1 (Chd1) was revealed to maintain open chromatin of pluripotentency and modulate cell reprogramming [59]. In the same year, chromodomain helicase DNA binding protein 1-like (Chd1l) was identified as a Parp1 associated protein in DNA damage response, and the macro-domain of Chd1l contains high affinity with PAR for chromatin recruitment [60,61]. Therefore, Chd1l is a candidate, containing similar function as Chd1, employed to remodel chromatin status in cell reprogramming. In our laboratory, Chd1l is explored as a modulator in cell reprogramming. Chromatin status of stemness gene loci is important to regulate gene activation, and Parp1 works as a guiding factor for recruiting Chd1l in cell reprogramming (unpublished data). Other ATP-chromatin remodeling enzymes, such as ino80, also modulate self-renewal and cell reprogramming [62]. In embryonic development, X chromosome inactivation is a process that regulates developmental gene expression. A histone H2A variant, MacroH2A, interacts with Parp1 and inhibits Parp1 activity by non-histone domain [13]. This interaction between MacroH2A and Parp1, therefore, inhibits the enzymatic activity of Parp1 in X-chromosome to silence gene expression. According to recent studies, pluripotent stem cells could be classed as having naïve and primed status, and only naïve pluripotent stem cells have the potential to form chimera. The difference between the naïve and primed statuses of stem cell is associated with X-chromosome reactivation [63,64]. Because activation of X-chromosome is important to change the status of pluripotent stem cells from primed to naïve, X-chromosome modulation by Parp1 could be investigated in reprogramming process [13].

## 4. Parp1 and Tumorogenesis

### 4.1. Parp1 Promotes Tumor Growth and Progression through Transcriptional Regulations

Tumorogenesis is a process caused by multifactorial-mediated cell transformation. These *in vivo* molecular events are explored in recent studies with animal models, including pharmacological inhibition and knockout of Parp1, and these results have helped to further understand the *in vivo* correlation between PAR and tumorogenesis. Chemo- or radio-therapy for cancer patients may cause DNA damage, and consequently induces programmed cell death. Suppression of genotoxic effects may help cancer cells to overcome programmed cell death. In an early study, Parp1 was identified in lymphoma cell line with enhanced expression level. Parp1 is crucial in eliciting the protection against genotoxic effects by modulating the DNA repair mechanism while it is activated by breaking DNA. In a study of Parp1 in DNA damage response, tumor suppressor p53 was one of the first proteins identified as a Parp1 interacting protein, and PARylation of p53 inhibited binding affinity of p53 for regulating gene expression during apoptosis program [65]. However, apoptosis sequentially activates caspase to cleave the N-terminus of Parp1 for blocking PARylation [20]. Parp1 deficient mice further reveal that Parp1 compromises p53 activation. Hence DNA damage increases mammary tumorigenesis. Parp1 also interacts with several proteins involved in DNA double strand break mechanism such as Xrcc1, DNA topoisomerases, and DNA-PK [66]. The interaction between Parp1 and associated proteins improves DNA repair and cell survival after genotoxic stress. Further, DNA repair mechanism of broken DNA also potentially provides accumulated mutation for tumor progression. In mesenchymal-epithelial transition (EMT), Snail-1, a transcription factor to modulate EMT gene expression, interacts with Parp1, and Parp1 mediated PARylation affects the stability of Snail-1 to regulate EMT process [27]. Smad3/4, a transcription factor activated by transforming growth factor beta (TGFβ) ligands, is also modulated by Parp1 through PARylation to attenuate Smad specific gene response in EMT process [67]. The opposite function of Parp1-mediated PARylation modulates the balance of transcription in cellular process.

### 4.2. Epigenetic Modulation of Parp1 in Tumorogenesis

Parts of Parp1 functions are to regulate gene expression by modulating transcription factor and epigenetic events in tumorogenesis. Aurora-B kinase is a chromosomal passenger protein in mitotic events through phosphorylation of histone H3 [68]. Parp1 interacts with Aurora-B kinase by BRCT domain and promotes PARylation of Aurora-B that suppresses the activity of Aurora-B kinase [69]. The specific inhibition of Aurora-B kinase could change epigenetic events through histone H3. HIF-1 activates hypoxia-dependent gene expression for tumor progression when tumor cells are cultured under hypoxic condition. Research has found depletion of Parp1 attenuates HIF-1 activation to cause necrosis and limit vascularization in leukemia cells [70]. Furthermore, Parp1 is important in modulating HIF-1α mediated transcription regulation: Parp1 directly interacts with HIF-1α to form a complex to influence the binding at its hypoxia elements. Aberrant hypermethylation of housekeeping gene promoters is observed to occur in cancer. Dnmt1 modulates DNA methylation in cancer, and Parp1 is identified to localize within the Dnmt1 promoter by PARylation [40]. This translocation of Parp1 to Dnmt1 maintains the unmethylated state of the *Dnmt1* promoter and enhances a genome-wild hypermethylation of housekeeping gene.

### 4.3. Use of Parp1 Inhibitor for Cancer Therapy

Parp’s inhibitors have been under investigation in recent years, and drugs that inhibit Parp1 activity have shown promise to treat BRCA1/2-assotiated cancers [71]. Parp1 inhibitors which are similar to NAD^+^ are designed to block catalytic domain while the DNA repair mechanism of Parp1 is associated with PARylation [22]. Because NAD^+^ is also an important metabolic substrate for other enzymes like Sirtuin 1 to maintain cellular function, most of Parp’s inhibitors always have strong side effects [72]. Recently, a specific inhibitor of Parp1 was developed by elucidating the crystal structure of Parp1. For instance, a potential Parp1 inhibitor, Veliparib (ABT-888), with oral bioavailability and the ability to cross the blood-brain barrier, has proven to suppress tumor progress in BRCA1 deletion and the BRCA2 mutation breast cancer xenograft model [73]. To date, Olaparib (AZD2281), Rucaparib (AG-014), BMN-637, CEP-9722, and Niraparib (MK4827) are the Parps inhibitors developing for clinical use which are under clinical trail from phase I to III [71,74].

## 5. Conclusions

**Figure 1:**
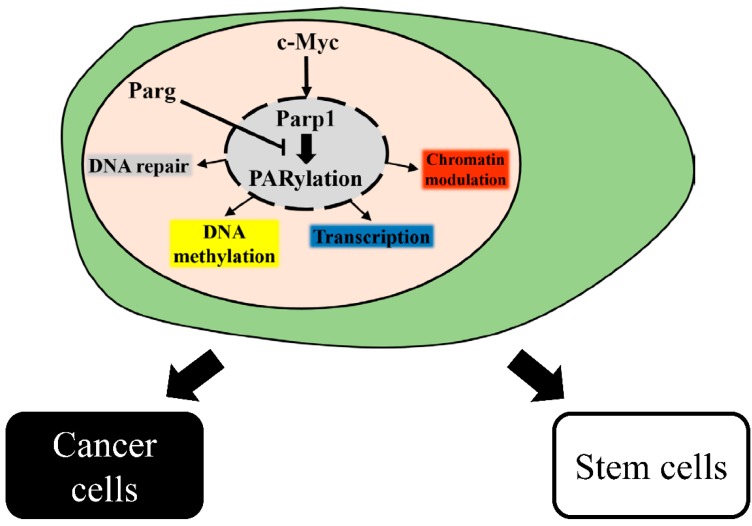
Intracellular regulations of Parp1. Parp1 is activated by c-Myc, Parp1 regulates cellular functions including DNA repair, transcription, DNA methylation, and chromatin modulation through PARylation of downstream proteins or directed binding to target protein. Poly(ADP-ribose) glycohydrolase (Parg) degrades the PAR chains of target protein to dismiss the downstream signaling. Precise regulation of the balance between PARylation and dePARylation is imperative to determine the cell fate of cells toward stem cell or cancer cell.

Parp1 is a key factor involved in many cellular processes through modulating nuclear events including transcription and epigenetic modulation (Table 1). In the steady status of somatic cells, the activity and expression level of Parp1 are required to maintain the cellular process. In cell reprogramming, Parp1 participates in complex mechanisms including transcription regulation, cell proliferation, and epigenetic remodeling to facilitate efficient reprogramming. Tumorigenesis, a multifactorial-mediated cell transformation, is a cellular process similar to cell reprogramming, and Parp1 assists in oncogene activation and provides resistance in chemotoxin for complete cell transformation (Figure 1). Finally, further exploring the functions of Parp1 in stem cells and in cell reprogramming will reveal the underlying mechanisms of tumor progression and help to develop a suitable treatment strategy for cancer therapy.

**Table 1:** Summary of PARP1-mediacted cellular regulations.

Cellular Function	Associated Protein	Regulation	Effect	Reference
DNA repair	p53	Interaction/PARylation	Negative	[4,65]
	Topoisomerases	Interaction	Positive	[66]
	Xrcc1	Interaction	Positive	[3]
	DNA ligIII	Interaction	Positive	[18]
	Ku70/80	Interaction/PARylation	Positive	[18,75]
	FACT complex	Interaction/PARylation	Positive	[18,28]
	Aurora-B kinase	Interaction/PARylation	Negative	[69]
Transcription	TFIIC	Interaction	Positive	[31]
	E2F1	Interaction	Positive	[11]
	CCN2	Modulation	Positive	[33]
	TCF4	Interaction	Positive	[37]
	Sox2	Interaction/PARylation	Negative	[47,48,49]
	Snail	Interaction/PARylation	Positive	[27]
	SMAD	Interaction/PARylation	Negative	[67]
	NRF1	Interaction/PARylation	Positive	[42]
	HIF1 alpha	Interaction	Positive	[70]
DNA methylation	CTCF	Interaction	Positive	[38,39]
	Dnmt1	Modulation	Positive	[39,40]
	Tip5	Interaction	Positive	[21]
Chromatin modulation	Histone H1	Modulation	Negative	[34,35]
	MacroH2A	Interaction	Negative	[13]
	Kdm5b	Interaction/PARylation	Negative	[34]
	Chd1l	Interaction	Positive	[60]
